# Loss of COX5B inhibits proliferation and promotes senescence via mitochondrial dysfunction in breast cancer

**DOI:** 10.18632/oncotarget.6222

**Published:** 2015-10-25

**Authors:** Shui-Ping Gao, He-Fen Sun, Hong-Lin Jiang, Liang-Dong Li, Xin Hu, Xiao-En Xu, Wei Jin

**Affiliations:** ^1^ Department of Breast Surgery, Key Laboratory of Breast Cancer in Shanghai, Collaborative Innovation Center for Cancer Medicine, Fudan University Shanghai Cancer Center, Shanghai, China; ^2^ Department of Oncology, Shanghai Medical College, Fudan University, Shanghai, China

**Keywords:** COX5B, proliferation, mitochondrial dysfunction, senescence, cytokine

## Abstract

COX5B, a peripheral subunit of the cytochrome c oxidase complex, has previously been reported to maintain the stability of this complex. However, its functions and mechanisms involved in breast cancer progression remain unclear. Here, by performing SILAC assays in breast cancer cell models and detecting COX5B expression in tissues, we found that COX5B expression was elevated in breast cancer. Down-regulation of COX5B in breast cancer cell lines can suppress cell proliferation and induced cell senescence which was accompanied by elevating production of IL-8 and other cytokines. Interestingly, conditioned medium from COX5B knockdown cells could promote breast cancer cell migration. Mechanistic studies reveal that COX5B silence induces an increase in production of ROS, depolarization of MMP and a decrease in ATP. What's more, silence of COX5B leads to metabolic disorders, such as increased glucose uptake and decreased lactate secretion. Collectively, our study shows that loss of COX5B induces mitochondrial dysfunction and subsequently leads to cell growth suppression and cell senescence. Cytokines such as IL-8 secreted by senescent cells may in turn alter the microenvironment which could enhance cell migration. These findings may provide a novel paradigm for the treatment which combined anti-cancer drugs with particular cytokine inhibitors such as IL-8 blockers.

## INTRODUCTION

Breast cancer is the most frequent cancer in women worldwide, accounting for 23% of total cancer cases and 14% of cancer deaths according to Global Cancer Statistics from 2011 [1]. Encouraging and advanced medical treatments that focus on systemic therapy and earlier diagnosis have been largely developed for breast cancer [2], however, many mechanisms underlying breast cancer progression are still not completely understood.

Mitochondria are the powerhouse of eukaryotic cells and are responsible for regulating energy metabolism, respiration and cell apoptosis [3]. Multiple investigators have demonstrated that mitochondria play an important role in cancers, and emerging evidences indicate that cancer cells are generally accompanied by mitochondrial dysfunction, such as the production of copious amounts of reactive oxygen species (ROS) or metabolic disorders [4–6]. High levels of ROS accumulation can promote DNA damage and genetic instability, which finally induce cell death and senescence [7]. Mitochondrial membrane potential has also been linked to an increase in malignant transformation [8]. Besides, metabolic changes are the hallmarks of cancer cells. Many cancer cells preferentially use glycolysis to generate ATP and metabolic intermediates, even in the presence of oxygen [9]. This preference is manifested by active glucose uptake and increased lactate production [10, 11]. Impaired mitochondrial metabolism subsequently contributed to the development of cancer progression [12]. Recent studies indicate that mitochondria proteins play a pivotal role in the respiration chain, and a loss of these proteins can induce mitochondrial dysfunction [13–15].

In our previous study, we conducted a SILAC (stable isotope labeling with amino acids in cell culture) assay in mammary epithelial cells (16N) and breast tumor cells (NT) that were both isolated from a single patient and found that COX5B was upregulated in breast cancer cells compared with normal cells [16]. COX5B is a peripheral nuclear-encoded subunit of CcO (cytochrome c oxidase), which is a multisubunit bigenomic protein complex that catalyzes the last step of the mitochondrial electron transport chain. Previous studies showed that the depletion of COX5B resulted in decreased CcO activity and suggested a regulatory role for COX5B [17]. However, few studies have reported the role of COX5B in human breast cancer. In this study, we first report that loss of COX5B inhibited cell proliferation and promoted cell senescence in breast cancer. We further explore the function and mechanism of COX5B in breast cancer.

## RESULTS

### COX5B is upregulated in breast cancer tissue and cell lines

**Figure 1:**
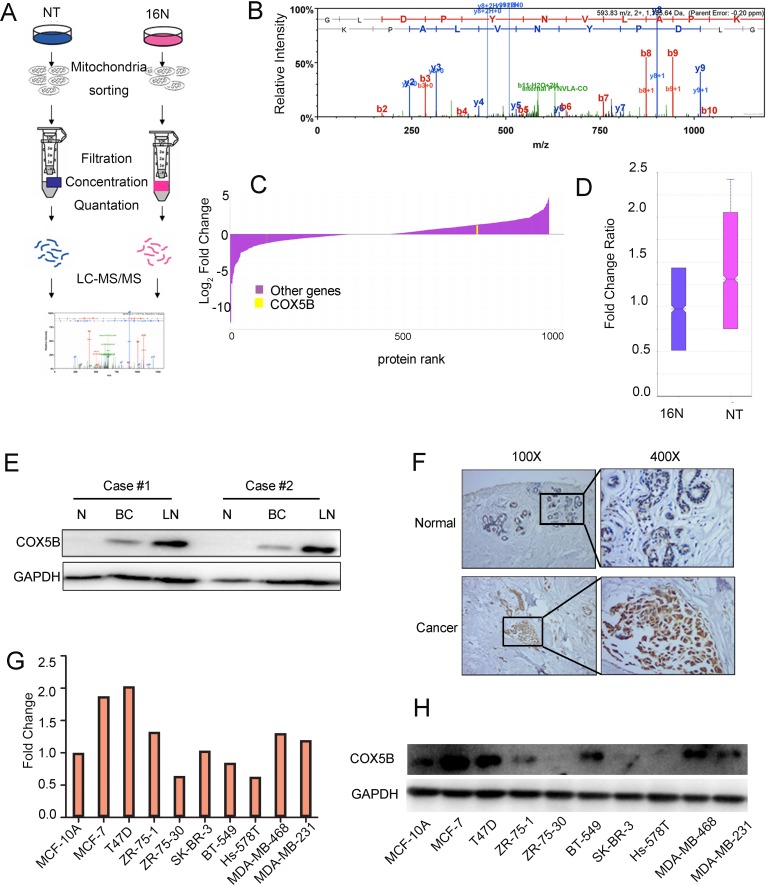
COX5B is upregulated in breast cancer tissues and cells (**A**) Schematic overview of quantitative stable isotope labelling by amino acids in cell culture (SILAC) and label-free mitochondrial proteomic analysis approaches. (**B**) The spectrum of COX5B obtained from MS. (**C**) The fold change of the total proteins from SILAC and the yellow line indicated the relative expression of COX5B (NT VS 16N). (**D**) The fold change ratio of the breast cancer cells (NT and 16N). (**E**) Expression of COX5B in two paired normal breast (N), breast cancer (BC) and lymph node (LN) tissues analyzed by Western blot. (**F**) Immunohistochemistry images of COX5B protein are shown in the large (400 × magnification) and small images (100 × magnification). (**G**) The mRNA level of COX5B expression in breast cancer cell lines. (**H**) The protein level of COX5B expression in breast cancer cell lines.

By performing stable isotope labeling with amino acids in cell culture (SILAC) in the normal epithelial cell line (16N) and primary breast tumor cell line (NT), we obtained many candidate genes, of which COX5B was increased in NT compared with 16N (Fig. 1A-1D). The peptide sequences of COX5B were also showed in Supplementary Figure S1. To validate the expression level of COX5B in breast cancer, we examined the expression of COX5B in two pairs of fresh, patient-derived tissue, including normal tissues, primary tumor tissues and metastatic lymph nodes. The results showed that COX5B was significantly increased in the two breast cancer samples compared with normal tissues, which was consistent with the results of SILAC (Fig. 1E). Interestingly, the COX5B expression was also elevated in metastatic lymph nodes. Furthermore, COX5B expression was examined in 40 tumor tissue and 20 normal or benign tissue samples. Upregulation of COX5B were detected in 26 of 40 samples, whereas 6 of 20 normal tissues with COX5B overexpression (Fig. 1F). In addition, COX5B was also upregulated in most breast cancer cell lines than the non-malignant cell line MCF10A, both at the mRNA and protein levels (Fig. 1G, 1H). The results indicated that COX5B was upregulated in breast cancer tissues and cell lines.

### Low expression of COX5B is associated with good prognosis

**Figure 2:**
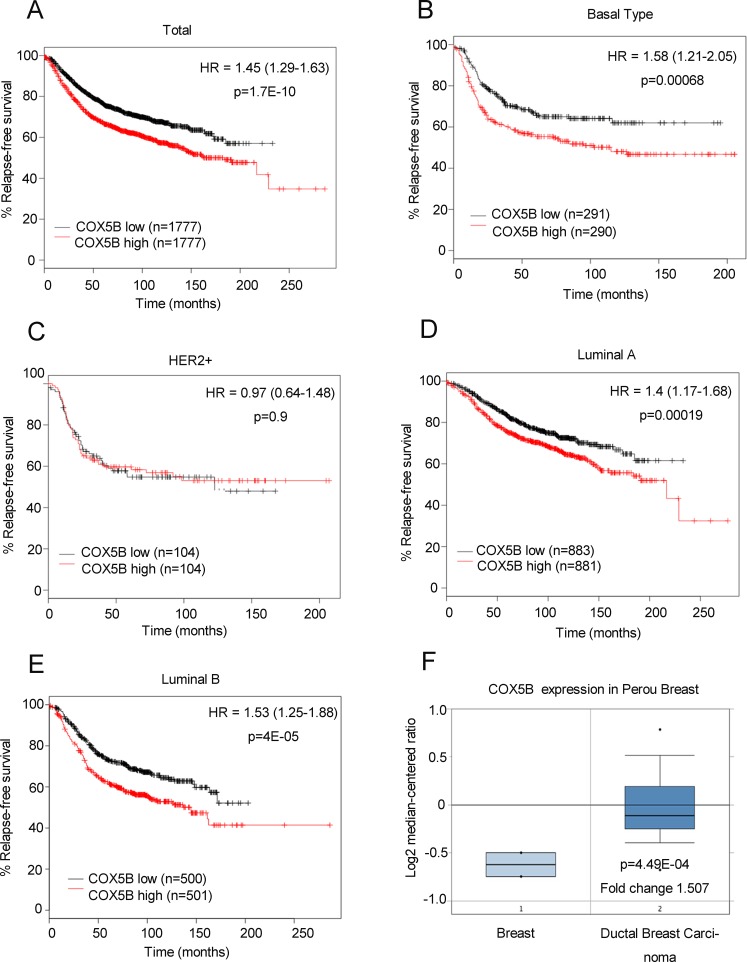
Low COX5B is associated with poor survival of breast cancer patients (**A-E**) Survival analysis of COX5B expression in patients with indicated subtype using the online Kaplan-Meier plotting tool (Total; Basal Type; Her2+; Lumina A; Lumina B). (F) Oncomine plots of COX5B expression pattern in human breast cancers vs normal tissues.

To further confirm the correlation between COX5B expression and clinical outcome, an online Kaplan-Meier plotting tool was employed to analyze survival according to Gyorffy. The results showed that low COX5B expression was associated with better DFS (disease-free survival) in patients with breast cancer (Fig. 2A). The similar results were also obtained in Luminal A, Luminal B and Basal subtypes, except for the HER2 subgroup (Fig. 2B-2E). We obtained the similar results when using the other probes of COX5B except the probe 213736_at in the Kaplan-Meier plotting tool (Fig S2-S4). We also utilized another online prediction tool, Oncomine, and found that COX5B expression was much higher in breast cancer tissues than in normal breast tissues (Fig. 2F). Taken all together, our findings suggested that low COX5B expression may indicate a good prognosis for breast cancer patients.

### Loss of COX5B inhibits breast cancer cell proliferation and induces cell senescence

**Figure 3:**
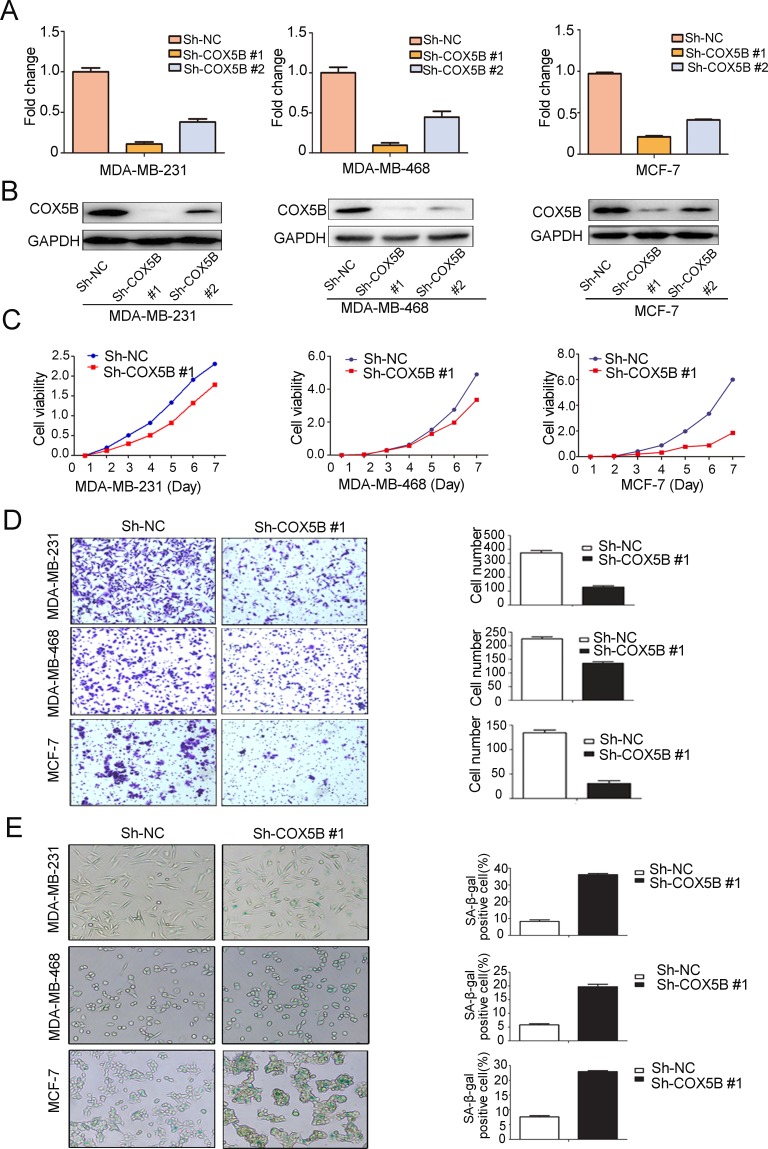
Loss of COX5B inhibits breast cancer cell proliferation and migration but induces cell senescence (**A**) The mRNA level of COX5B after its stable knockdown in MDA-MB-231, MDA-MB-468, and MCF-7 cells by Real-time PCR. (**B**) The expression of COX5B protein in three stably knocked down cell lines by Western blot. (**C**) The proliferation of three stably knocked down cell lines. (**D**) The migration ability of each cell line was evaluated with a transwell assay *in vitro*. The left panel shows photos of representative fields (100 × magnifications) of migratory cells, and the right panel shows histograms of the results. (**E**) Representative images of β-galactosidase staining assay (left) and positive cell quantification (right) showed a marked increment in three stably knocked down cell lines. The statistical analysis was performed using Student's *t*-test (n=3) (*P* < 0.05).

To assess COX5B function in breast cancer progression, we stably knocked down COX5B in 3 breast cancer cell lines (MDA-MB-231, MDA-MB-468 and MCF-7). The efficiency of knockdown was confirmed with mRNA and protein level analyses (Fig. 3A, 3B). We then explored the effects of COX5B depletion on cell proliferation and migration. The result showed that loss of COX5B inhibited both cell proliferation and migration in the three cell lines (Fig. 3C, 3D). In the process, cells with sh-COX5B became flattened and elongated after three passages, which were morphological transformation of cell senescence. Thus, a β-galactosidase staining assay was used to determine whether the cells undergo cell senescence. We found that more COX5B-depleted cells (25~45%) stained positively than control cells (7~16%) (Fig. 3E), suggesting that loss of COX5B induced senescence.

### Loss of COX5B enhanced IL-8 and other associated cytokines releasing

**Figure 4:**
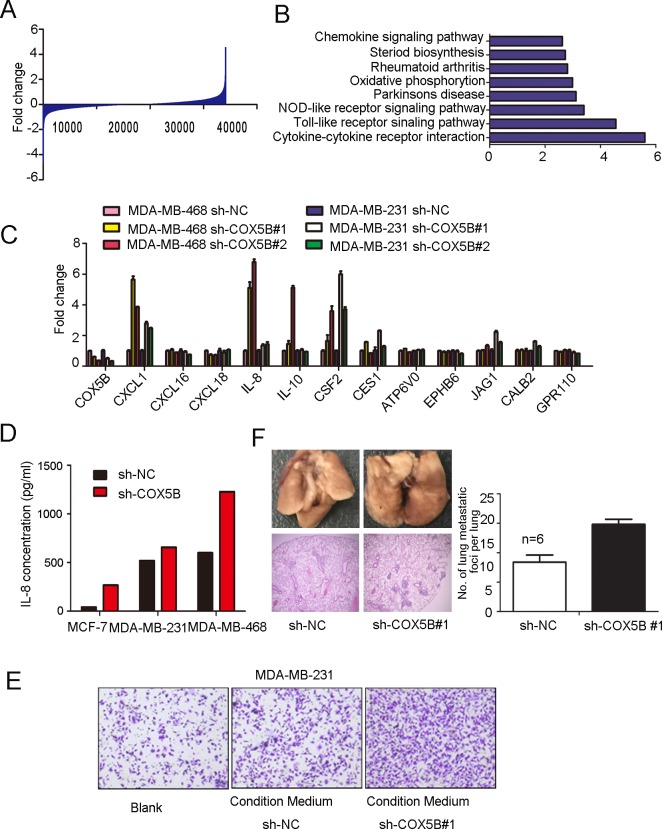
Loss of COX5B enhanced IL-8 and other associated cytokines releasing The entire genes obtained from microarray profile assay. (**B**) GO enrichment analyses of COX5B knockdown and control cells. (**C**) Real-time PCR validated the candidate genes. (**D**) IL-8 secretion analyzed by ELISA. (**E**) Migration assay of MDA-MB-231 induced by MDA-MB-231 sh-COX5B #1- and sh-NC-conditioned medium. (**F**) Left panel shows representative images for the lung metastatic nodules, the upper two photos show number of metastatic nodules in lungs surface indicated by red arrows and the lower ones display hematoxylin-eosin stained sections of lungs (magnification ×40). Right panel, quantification of the number of lung metastatic foci per lung, the number of foci was counted in three sections per lung under the microscope n=6 *p*<0.05.

In regard to the mechanisms by which loss of COX5B inhibited cell movement and induced cell senescence, microarray expression profile analysis were performed and revealed about 1000 genes changing at least 2-fold expression in response to COX5B down-regulation (Fig. 4A), of which are involved in various pathways, including the “chemokine signal”, “oxidative phosphorylation”, “NOD-like receptor signaling”, “Toll-like receptor signaling” and “cytokine-cytokine receptor interaction” pathways, through GO enrichment analyses (Fig. 4B).

We then picked genes showing the most statistically significant expression differences, including both up- and down-regulated genes, to validate by real-time PCR analysis in MDA-MB-468 and MDA-MB-231 cell lines. Six of these showed validated expression differences by this method: CXCL1, CXCL16, IL-8, IL-10, CSF2, CES1 (Fig. 4C). It had been widely demonstrated that IL-8 could alter the microenvironment to impact cell migration, we then detected IL-8 secretion with ELISA assay and the results were consistent with the mRNA expression data (Fig. 4D). To evaluate whether the upregulation of cytokines may influence cell migration, transwell assay was performed in MDA-MB-231 cells, with sh-COX5B and sh-NC cells cultured medium as chemoattractant in the lower chamber. We found that condition medium from COX5B knockdown cells promoted cell migration (Fig. 4E). To further explore the metastatic ability of COX5B shRNA stable cancer cell line in the xenograft tumor model, two groups of mice were equally injected with sh-NC or sh-COX5B cells vis tail vein. The result showed that mice with sh-COX5B group increase the average number of metastatic lung foci compare with sh-NC group (Fig. 4F).

### Loss of COX5B leads to mitochondrial dysfunction

Given that COX5B is a subunit of cytochrome c oxidase and plays a crucial role in the mitochondrial electron transport chain, we further evaluated the effect of COX5B on mitochondrial metabolic function in stable knockdown cell lines.

**Figure 5:**
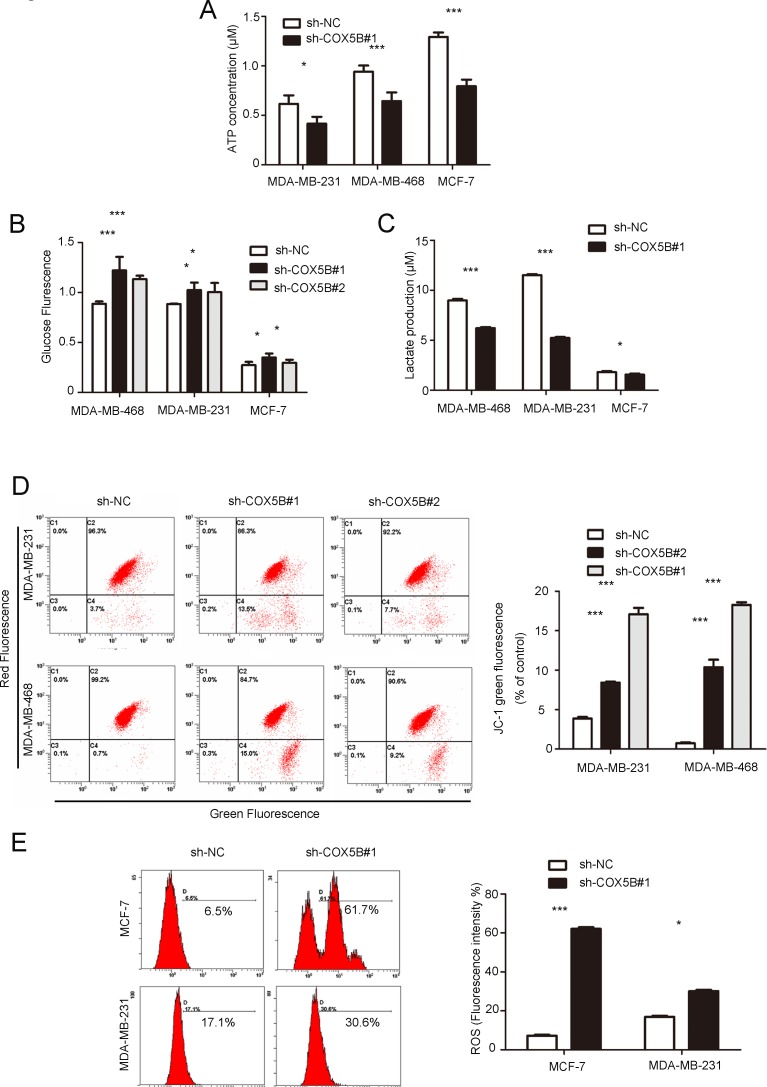
Down-regulation of COX5B leads to mitochondrial dysfunction (**A**) ATP production in COX5B-depleted and control MDA-MB-231, MDA-MB-231, and MCF-7 cells. (**B**, **C**) Glucose uptake and lactate secretion were detected in COX5B knockdown or control MDA-MB-231, MDA-MB-231, and MCF-7 cells. (**D**) FACS analysis of mitochondrial membrane potential. The left panel shows a representative graph of the indicated cells, the right panel shows statistical data (**E**) FACS analysis (left) and Statistical data (right) of mitochondrial ROS generation in indicated cells. Data were presented as mean ± SD from three independent experiments. *P < 0.05, ***P < 0.001 compared with control.

Intracellular ATP measurement indicated that COX5B knockdown significantly decreased the levels of cellular ATP compared with the control group (Fig. 5A). Because the source of ATP is dependent on the processes of aerobic respiration and anaerobic respiration, we further detected the glucose uptake and lactate secretion to assess the metabolic pathway. The results showed that glucose uptake was slightly elevated in response to COX5B depletion compared with the control group (Fig. 5B). However, lactate secretion decreased in the COX5B down-regulation group (Fig. 5C). In addition, down-regulation of COX5B resulted in a significant loss of mitochondrial potential and increase in ROS production (Fig. 5D, 5E). Taken together, these results suggested that COX5B knockdown promoted aerobic respiration and led to internal mitochondrial dysfunction, which may be associated with cell senescence and cytokine increase.

## DISCUSSION

Breast cancer is a multifaceted disease with a variety of biological subtypes. Breast cancer relapse remains an obstacle in successful treatment. Recently, selective targeting of breast cancer by some small molecules may hold promise in a subset of patients [18]. However, more biomarkers and targets for the management of this disease are still needed.

Our work revealed that COX5B was overexpressed in human breast cancer tissues and cell lines, in line with this, Chen et al. have reported that COX5B expression was elevated in both MCF-7 and MDA-MB-231 cells compared with MCF-10A cells (normal epithelial cell) by proteomic analysis [19]. Coincidentally, COX5B upregulation was also reported in prostate cancer and cutaneous squamous cell carcinoma [20, 21]. In our study, we further validated that COX5B expression was higher in primary tumor tissues and metastatic lymph nodes than in normal tissue. In addition, a high expression of COX5B predicted a poor prognosis for breast cancer patients based on analyses using the Oncomine database and a Kaplan Meier plot. These results indicated that COX5B was associated with poor prognosis.

Considering the role COX5B in mitochondrial electron transport chain, COX5B knockdown may be associated with mitochondrial dysfunction and metabolic disorder. Indeed, our results supported that COX5B-depletion induced mitochondrial dysfunction by increasing ROS production and decreasing MMP depolarization and intracellular ATP generation. Previous studies reported that mitochondrial dysfunction was associated with senescence [22]. Besides, mitochondrial ROS and mitochondrial membrane potential (MMP) are involved in senescence-associated retrograde signaling [23, 24]. Emerging evidence indicates that glucose metabolism also shifts in senescent cells [25–27]. Taken together, the mitochondrial dysfunction and metabolic disorder may play vital role in cell senescence.

In our study, we also found that loss of COX5B induced senescence and increased multiple cytokines secretion such as IL-8, CXCL1, CSF2 and most of which were enriched in cytokine-cytokine receptor interaction pathway. Previous studies have predicted that multiple cytokines or growth factors were involved in the tumor progression of metastasis [9, 28]. For example, IL-8 expression was associated with pathologic stage, high tumor grade and metastatic property in prostate cancer [29] and breast cancer [30, 31]. What's more, IL-8 was also correlated with an increased vascularization in gastric carcinoma [32]. CXCL1 also plays a vital role in multiple tumor progression. It was reported that increased CXCL1 protein levels were associated with higher-grade stage in prostate cancer [33], advanced tumor stage and poorer prognosis in gastric cancer through VEGF signaling. Those studies suggested that IL-8 or CXCL1 might be potent to improve anti-angiogenic therapy for multiple cancers [34].

However we didn't probe deep into the linkage between COX5B silence and these cytokines expression. This linkage may be associated with MAVS (mitochondrial antiviral signaling) pathway and excessive activation of MAVS-mediated antiviral signaling leads to dysfunction of mitochondrion [35]. COX5B was validated to reduce MAVS signaling by repressing ROS production. These alterations may induce cell endoplasmic reticulum stress and finally lead to cytokines secretion. Taken together, cytokines secretion induced by loss of COX5B may be associated activation of MAVS signaling.

Recent research reported that certain chemotherapy or radiotherapy agents can also induce cell senescence. However, clinical treatment may also induce cell cytokines secretion. In brief, these findings suggested that anti-cancer regimes may not only suppress the proliferation of target cells but also alter its microenvironment. Therefore, clinical performance should combine anti-cancer drugs with inflammation inhibitors such as IL-8 blockers.

Loss of COX5B could inhibit cancer cell proliferation and lead to mitochondrial dysfunction. It is very interesting to understand whether those phenomena are tumor specific. In our study, we found that knockdown of COX5B in MCF10A (breast epithelial cell) can slightly inhibit cell proliferation and elevated the expression of IL-8. However, loss of COX5B in MCF10A only made a little decrease in depolarization of MMP (25 folds change in MDA-MB-468 vs 1.7 in MCF10A) and didn't alter the production of ROS (9.5 folds change in MCF7 vs 1.15 in MCF10A). Those results suggested that breast cancer cell was more sensitive to COX5B depletion than MCF10A cells (Figure S5).

In summary, we identified that COX5B was upregulated in breast cancer tissue and cell lines, which was associated with poor prognosis. Furthermore, loss of COX5B inhibited proliferation and induced the senescence and SASP. The mechanisms might be associated with mitochondrial dysfunction with increased ROS production and decreased MMP and metabolism disorder. However, the precise mechanism remained unclear and needed to be further explored. Indeed, loss of COX5B may have different functions on target cells and the surrounding environment. These findings may provide a new perspective for combined treatment of anti-cancer agents with inflammation inhibitors such as IL-8 blockers for breast cancer patients.

## MATERIALS AND METHODS

### Cell culture and breast tumor specimens

HEK-293T and the breast cancer cell lines MDA-MB-231, MDA-MB-468, and MCF-7 were obtained from the American Type Culture Collection (ATCC) and maintained under conditions specified by the provider. The 16N (mammary epithelial) and NT (breast tumor) cells were separated from a patient with breast cancer. All cells were cultured in a 5% CO_2_ incubator at 37°C.

Human breast cancers specimens were obtained from patients undergoing surgery for breast cancer at the Shanghai Cancer Center. All patients provided written informed consent and the study was approved by the Ethics Committee of the Cancer center of Fudan University

### shRNA transfection and virus packaging

The sh-COX5B and sh-NC (blank vector) plasmids were purchased by GeneChem (GeneChem). The target sequences were as follows: 5′-CTGGGTTGGAGAGGGAGAT-3′(sh-COX5B#1); 5′-GCTGTGGAGCCCATTACAA-3′ (sh-COX5B#2). The plasmids were transfected into 293T cells using Lipofectamine 2000 (Invitrogen) according to the manufacturer's instructions. Virus-containing medium was collected 48 h after the transfection of 293T cells and added to the cancer cells.

### RNA isolation and Real-time PCR

Total RNA was purified using TRIzol Reagent (Invitrogen Life Technologies). Equivalent amounts of cDNA were synthesized for each sample using Reverse Transcriptase Reagents (TakaRa) according to manufacturer's instructions. Real-time PCR was performed on an ABI Prism 7900HT detection system using the Premix ExTaq perfect real-time system (TakaRa) with gene-specific primer pairs. Quantitation was performed using the ΔΔCt method, and GAPDH expression used as an internal reference. Each sample was given in three replicates. All used primer sequences were shown in Supplementary Table 1.

### Protein extraction and Western blot analysis

Whole-cell lysates were obtained using Pierce T-PER (Tissue Protein Extraction Reagent; Thermo Fisher Scientific Inc.), which contained protease inhibitor cocktail tablets (Roche). In total, 30 μg of the cell lysate was subjected to SDS-PAGE and transferred to PVDF membranes (Pall). The membranes were blocked in 5% milk for 1 h and then washed with Tris-Buffered Saline (TBS) containing 0.1% Tween-20 and incubated with primary antibodies within 2 h (COX5B 1:1000, GAPDH 1:3000, protein tech), followed by incubation with HRP-conjugated secondary antibodies for 1 h. The immunoreactive bands were identified using enhanced chemiluminescence.

### Cell proliferation

Cell viability was determined using Cell Counting Kit-8 (Sigma-Aldrich). The cells were seeded on 96-well plates at a density of 2 × 10^3^ cells/well and then every 24 h incubated with CCK-8 agent for 3 h at 37°C for 7 days. The cell viability signal was detected at 450 nm using a microplate reader (Tecan Sunrise, Switzerland).

### β-galactosidase staining

The cells were stained using a cellular senescence β-galactosidase staining kit (Beyotime) strictly according to the manufacturer's recommendation. The blue stained cells were detected every 2 h for 12 h. At least 5 representative fields were randomly selected to quantitate the percentage of SA-β-gal-positive cells.

### Cell migration assays

Cells (5 × 10^4^) were plated in the top chamber of transwell chambers (BD Biosciences) in media without FBS. Medium with 20% FBS was used as a chemoattractant in the lower chamber. The cells were incubated for 15-20 h, then removed the cells that did not migrate. The cells on the lower surface of the membrane were stained with methanol and 0.25% crystal violet and five fields of each membrane should be counted for each sample.

As for conditioned transwell, cells (3 × 10^5^) were seeded in 6-well plate. After cell adherence overnight, the cells were washed three times with PBS and refreshed with 1.2 ml FBS-free DMEM culture medium for 36 h. The supernatant of each sample was collected and added in lower chamber as attractants, cell suspension solution without FBS was placed in upper chamber. The remaining steps were in line with above.

### ATP assay

Cells were harvested, washed, and lysed in ATP buffer. The ATP standard solutions and samples were added to a 96-well plate involving several other agents according to ATP assay kit (BioVision). Then all wells were incubated for 30 min and measured at 570 nm by a microplate reader (Tecan Sunrise)

### Mitochondrial ROS determination

Cells (5 × 10^5^) were harvested, washed and resuspended in (Invitrogen Corp.) working solution with 5 μM MitoSOX for 15 min at 37°C. After centrifuging, cells were resuspended in 500 μL PBS and analyzed for mitochondria ROS production by ﬂow cytometry (BD Biosciences).

### Mitochondria membrane potential (MMP)

Cells of 10^6^ were washed, resuspended and incubated at 37°C for 15 min using MMP detection kit (Yisheng). Cells were analyzed using a BD FACS flow cytometer. Loss of mitochondrial ΔΨm is presented as the relative ratio of green to red fluorescence.

### Glucose uptake assay

The cells were seeded at a density of 2 × 10^4^ cells/well in 96-well black, clear bottom plate (Corning) and disposed by Glucose Uptake Cell-Based Assay Kit (Cayman) according to the manufacturer's operations. Cells were measured using a plate reader (excitation/emission=485 nm/535 nm).

### Lactate production assay

A total of 3 × 10^5^ cells were cultured in a 6-well plate for 72 h. Supernatant was collected and was deproteinized due to high LDH content. Supernatant was collected and measured by lactate assay kit from BioVision (Mountain View).

### ELISA

Supernatants from sh-COX5B or sh-NC cells were collected respectively and centrifuged at 400 × g for 5 min at 4°C, then performed using human IL-8 ELISA kit (Neobioscience) following the manufacturer's instructions. The absorbance was finally monitored at 450 nm microplate reader.

### Transcriptome Microarray Analysis

Total RNA was submitted to Bioassay Laboratory of CapitalBio Corporation (CapitalBio) for gene expression profiling using the Agilent Human Gene Expression. Candidate genes were defined as 2.0 folds expression change.

### *In vivo* tumor metastasis assay

MDA-MB-231 cells treated with sh-NC or sh-COX5B virus were suspended in PBS to an appropriate concentration (2 × 10^6^ cells/mL) and mice were injected with 0.2 mL cell suspension via the tail vein (6 mice/group). After about 2 months, the mice were sacrificed and the lungs were removed and fixed in 4% paraformaldehyde in phosphate-buffered saline overnight and subsequently embedded in paraffin wax. Sections were cut at a thickness of 4 μm and stained with hematoxylin and eosin for histological analysis. The number of tumor metastatic nodules was counted under a dissecting microscope.

### Immunohistochemistry

The tumors were dewaxed, hydrated, and the endogenous peroxidase activity was quenched. After antigen retrieval, the tissue were blocked and incubated with primary antibody and secondary antibody. Staining was visualized using colorimetric detection with 3, 3-diaminobenzidine. For semi-quantitative classes were used to describe staining intensity (1, weak; 2, moderate; and 3, intense) and the staining distribution was determined a value from 0 to 4 as follows: 0, <5%; 1, 5%-25%; 2, 25%-50%; 3, 50%-75%; and 4, >75%. The staining distribution and the staining intensity were multiplied for each case. Tissues with an immunohistochemical score of 3 or less were considered to have low expression, and those with a score of 4 to 12 were considered to have high expression.

### Kaplan-Meier plotter analysis

The Kaplan-Meier survival analysis was performed as previously descripted. COX5B disease-free survival curve were analyzed by probe 202343_x_at.

### Statistical analysis

The means ± the standard deviation (S.D.) were calculated and presented for each data point. Statistical analyses were performed using a paired Student's *t* test. For all experiments, *P* values < 0.05 were considered statistically significant.

## SUPPLEMENTARY MATERIAL FIGURES AND TABLE


