# Supplementary material for: Loss of COX5B inhibits proliferation and promotes senescence via mitochondrial dysfunction in breast cancer
Source: Oncotarget. 2015 Oct 25;6(41):43363–74. doi: 10.18632/oncotarget.6222 (PMC4791237; doi:10.18632/oncotarget.6222)
Supplement: Supplementary file 1 [file oncotarget-06-43363-s001.pdf]

Loss of COX5B inhibits proliferation and promotes senescence via mitochondrial dysfunction in breast cancer

Supplementary Material

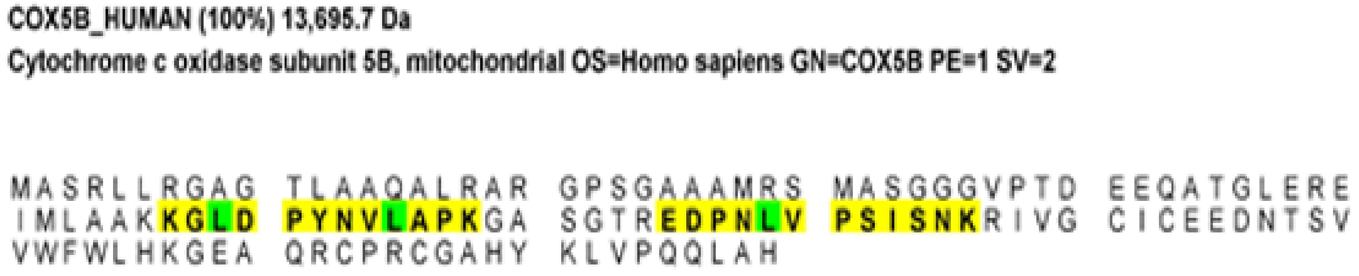

Figure S1: The peptide sequences of COX5B

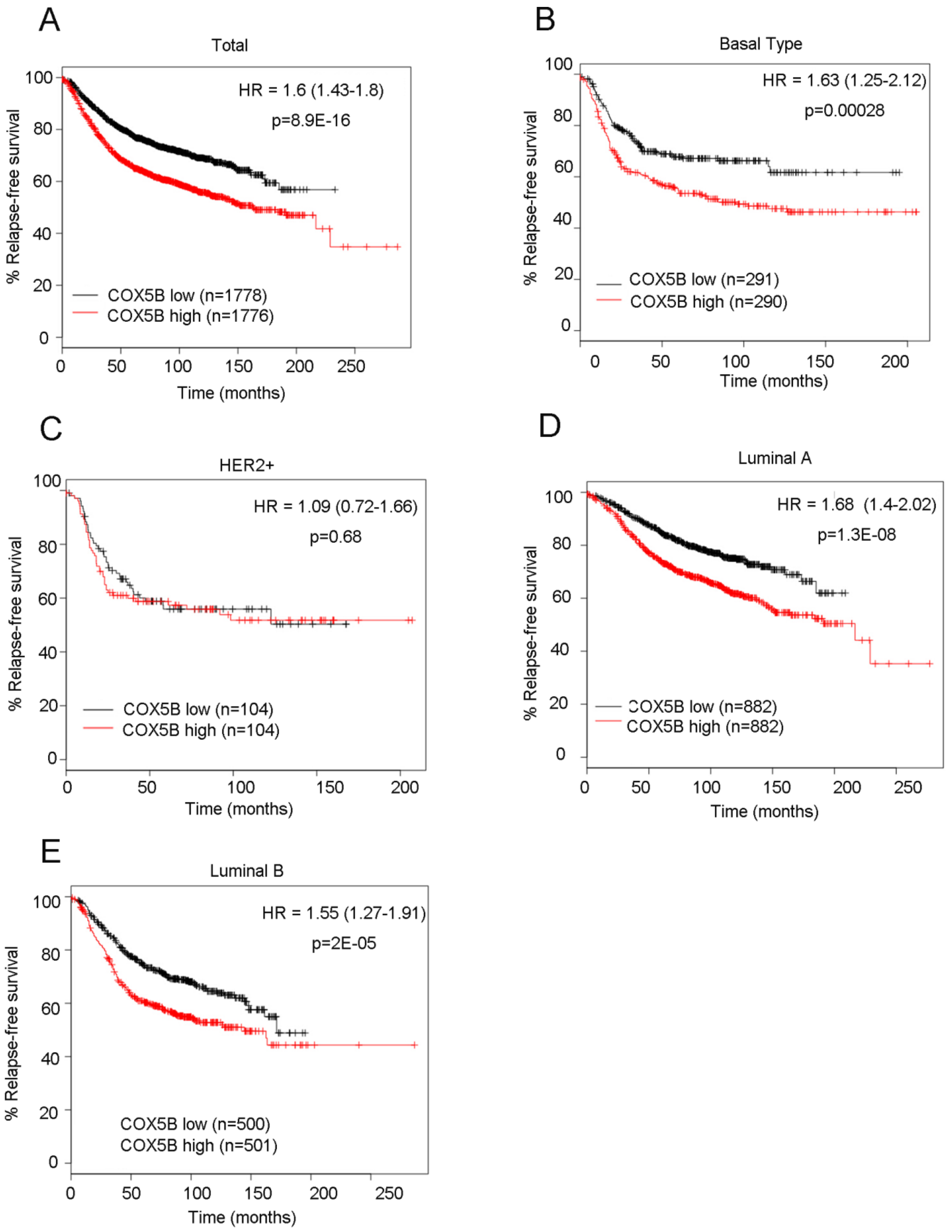
A
Total
HR = 1.6 (1.43-1.8)
p=8.9E-16
COX5B low (n=1778)
COX5B high (n=1776)
% Relapse-free survival
Time (months)
B
Basal Type
HR = 1.63 (1.25-2.12)
p=0.00028
COX5B low (n=291)
COX5B high (n=290)
% Relapse-free survival
Time (months)
C
HER2+
HR = 1.09 (0.72-1.66)
p=0.68
COX5B low (n=104)
COX5B high (n=104)
% Relapse-free survival
Time (months)
D
Luminal A
HR = 1.68 (1.4-2.02)
p=1.3E-08
COX5B low (n=882)
COX5B high (n=882)
% Relapse-free survival
Time (months)
E
Luminal B
HR = 1.55 (1.27-1.91)
p=2E-05
COX5B low (n=500)
COX5B high (n=501)
% Relapse-free survival
Time (months)

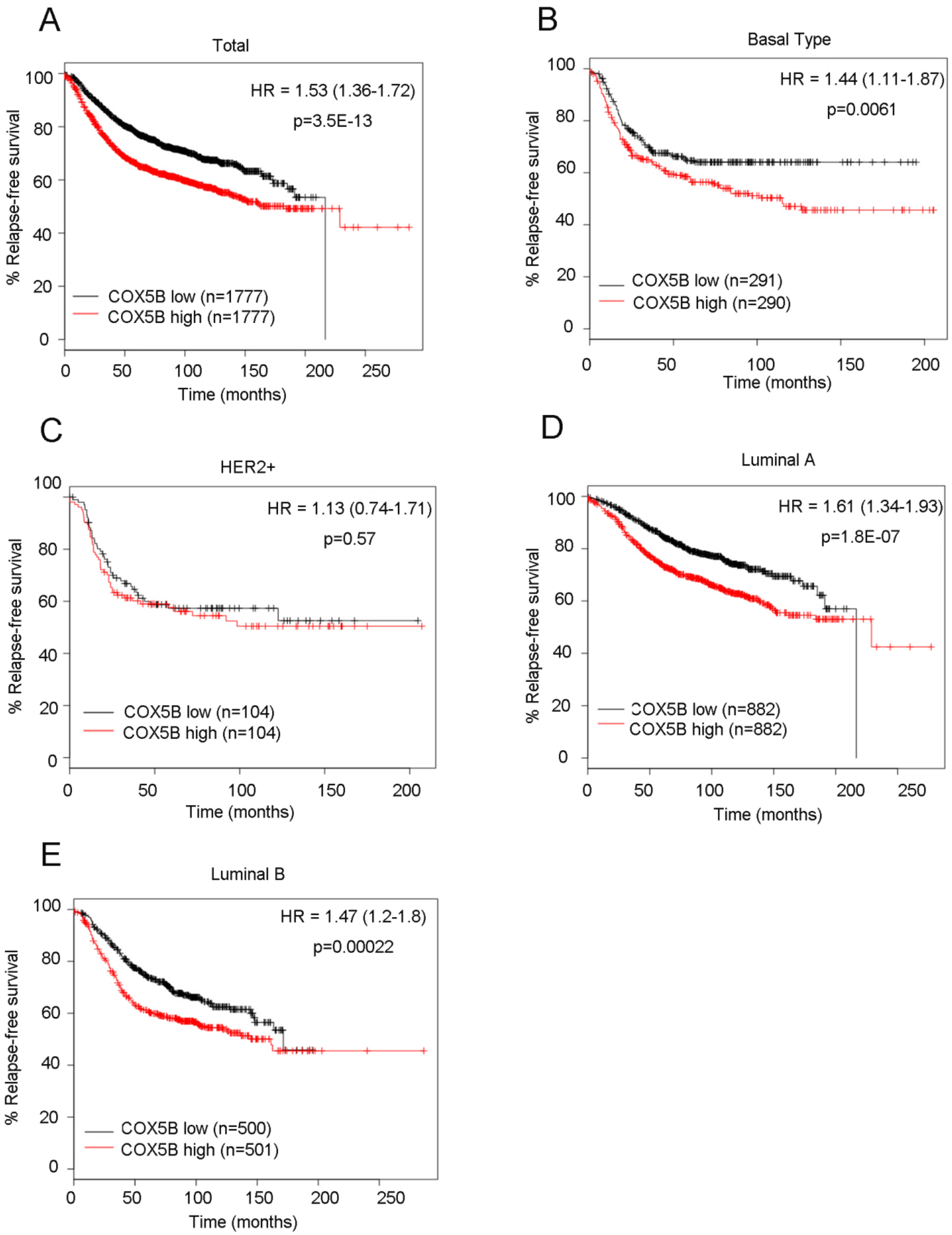
A
Total
HR = 1.53 (1.36-1.72)
p=3.5E-13
% Relapse-free survival
COX5B low (n=1777)
COX5B high (n=1777)
Time (months)
B
Basal Type
HR = 1.44 (1.11-1.87)
p=0.0061
% Relapse-free survival
COX5B low (n=291)
COX5B high (n=290)
Time (months)
C
HER2+
HR = 1.13 (0.74-1.71)
p=0.57
% Relapse-free survival
COX5B low (n=104)
COX5B high (n=104)
Time (months)
D
Luminal A
HR = 1.61 (1.34-1.93)
p=1.8E-07
% Relapse-free survival
COX5B low (n=882)
COX5B high (n=882)
Time (months)
E
Luminal B
HR = 1.47 (1.2-1.8)
p=0.00022
% Relapse-free survival
COX5B low (n=500)
COX5B high (n=501)
Time (months)

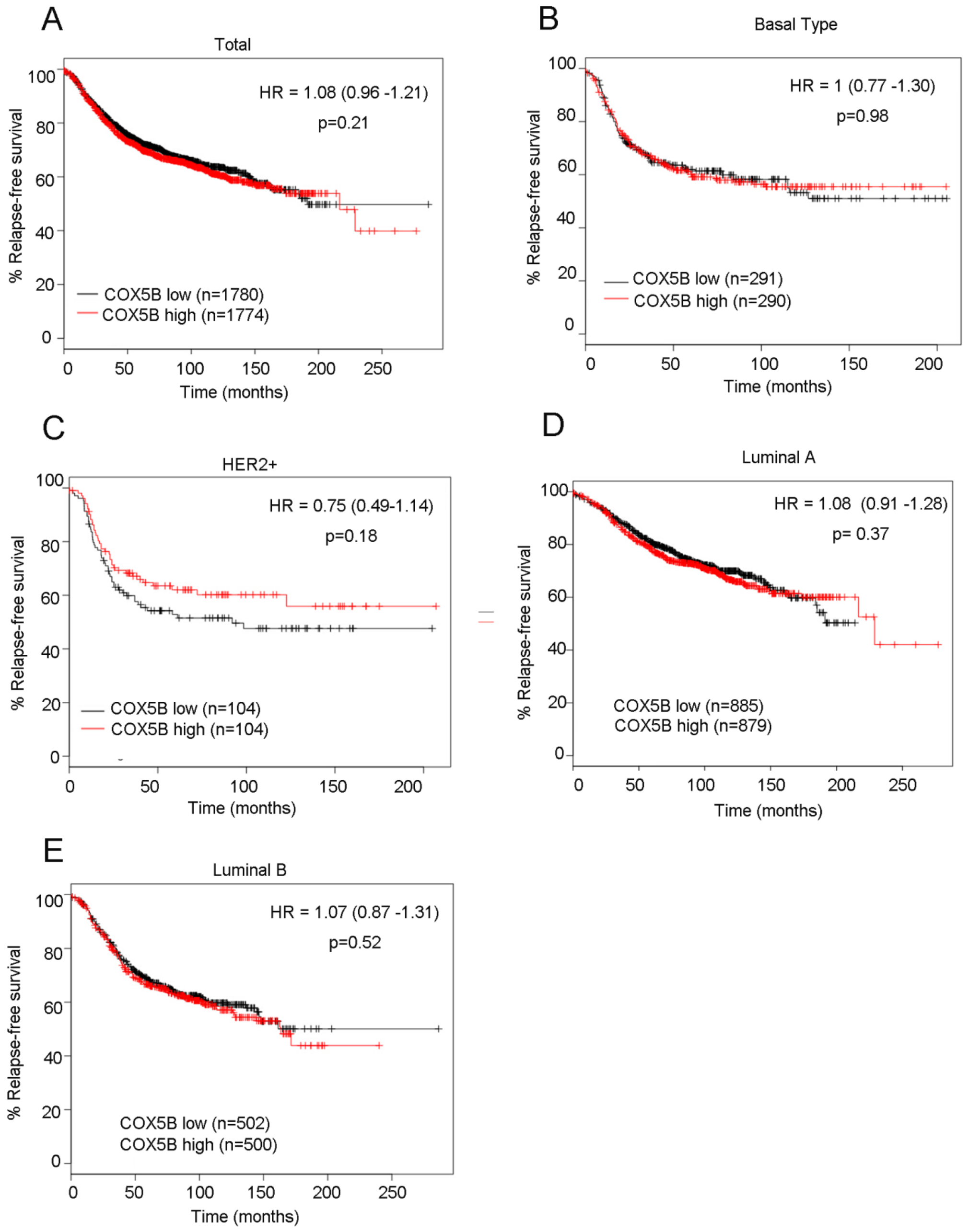

Supplementary Figure S2-4

The survival analysis of COX5B expression in patients with indicated subtype with other probes of COX5B (211025_x_at, 213735_s_at, 213736_at) using the online Kaplan-Meier plotting tool (Total; Basal Type; Her2+; Lumina A; Lumina B)

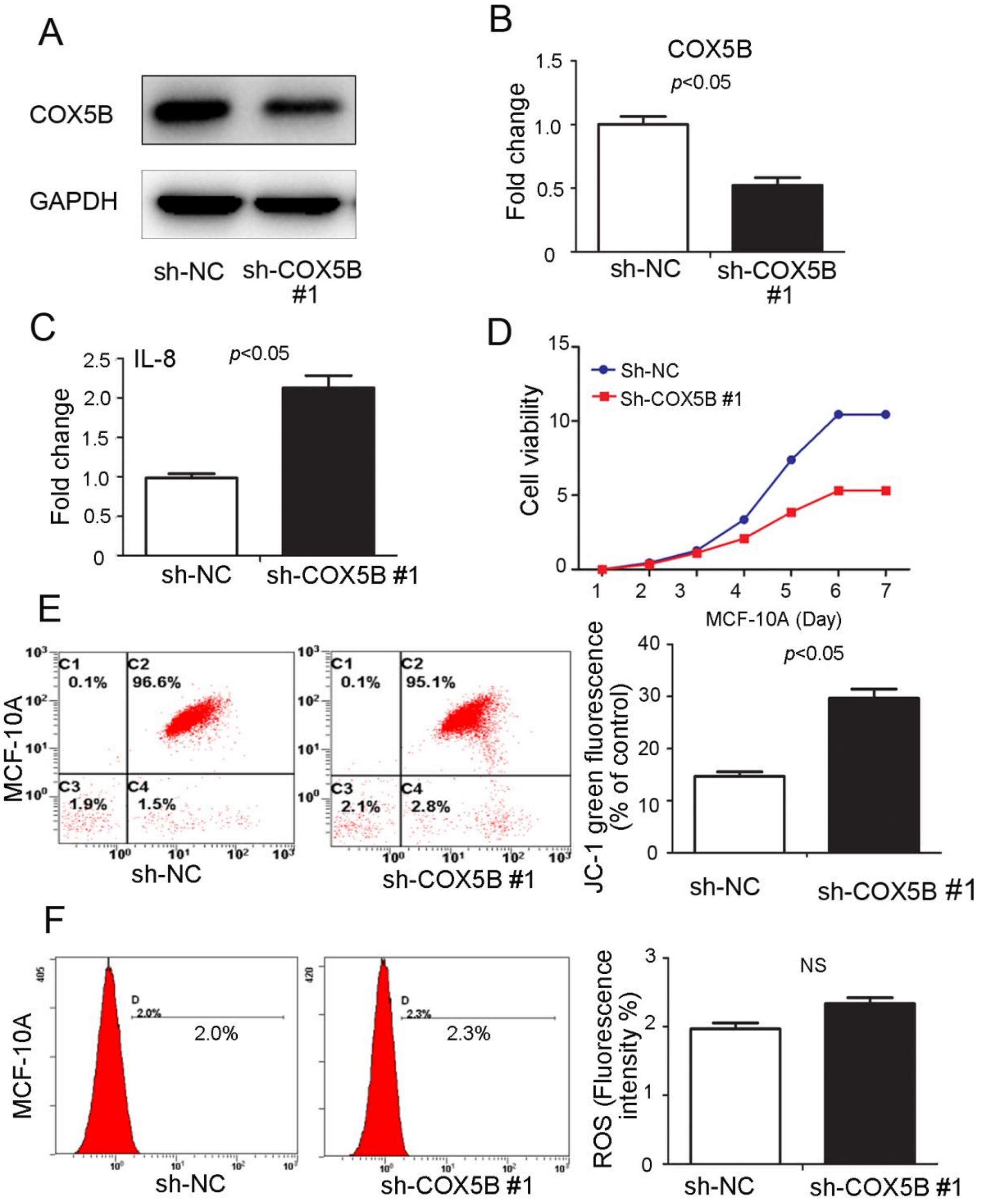

Supplementary Figure S5 Down-regulation of COX5B in MCF10A

(A B) The protein and mRNA expression of COX5B in MCF10A (sh-NC and sh-COX5B #1). (C) IL-8 expression analyzed by Real-time PCR. (D) The proliferation of MCF10A by CCK8. (E) The depolarization of MMP of MCF10A. (F) FACS analysis of mitochondrial ROS.

Supplementary Table 1: Primer sequences

Gene name	primer	sequence
COX5B	Foward	5’-GTTTTGGCTGCACAAAGGGC-3’
	Reverse	5’-CTGGGGCACCAGCTTGTAAT-3’
CXCL1	Foward	5’-TCGAAAAGATGCTGAACAGTGAC-3’
	Reverse	5’-CCAGATTGAACTAACTTGGGGTTG-3’
CXCL16	Foward	5’-ATGCTTACTCGGGGATTGTG-3’
	Reverse	5’-GTGGTTTCATTGGGACGAGT-3’
CXCL18	Foward	5’-AGTGGCTCACGCCTGTAATC-3’
	Reverse	5’-TCTACCTCCGGAGTGCAAGT-3’
IL-8	Foward	5’-AGGGTTGCCAGATGCAATAC-3’
	Reverse	5’-CCTTGGCCTCAATTTTGCTA-3’
IL-10	Foward	5’-CCACGTGTTGAGATCATTGCT-3’
	Reverse	5’-TGCATCGATTTTGCTCCCCT-3’
CSF2	Foward	5’-GCGTCTCCTGAACCTGAGTAG-3’
	Reverse	5’-TCGGCTCCTGGAGGTCAAAC-3’
CES1	Foward	5’-AAGCAGGAGTTTGGCTGGTT-3’
	Reverse	5’-GCTCCAGCATCTCTGTGGTT-3’
ATP6V0	Foward	5’-TGGCACTGAATTGAGCAAAG-3’
	Reverse	5’-TCACGCTCGTAAAACACGTC-3’
EPHB6	Foward	5’-CAGCCTAGAAGACCTGCCTG-3’
	Reverse	5’-CCATCGGAGGGTTGGGTTTG-3’
JAG1	Foward	5’-TGTGAAATTGCTGAGCACGC-3’
	Reverse	5’-ACACAAGGTTTGGCCTCACA-3’
CALB2	Foward	5’-ATGGCAAATTGGGCCTCTCA-3’
	Reverse	5’-GCCGCTTCTATCCTTGTCGT-3’
GPR110	Foward	5’-CTGGAAAGGGATTCCAGTGA-3’
	Reverse	5’-GGTCCATTGACCTGAGCATT-3’
GAPDH	Foward	5’-GGTGGTCTCCTCTGACTTCAACA-3’
	Reverse	5’-GTTGCTGTAGCCAAATTCGTTGT-3’
